# Pharmacological Induction of Heme Oxygenase-1 Impairs Nuclear Accumulation of Herpes Simplex Virus Capsids upon Infection

**DOI:** 10.3389/fmicb.2017.02108

**Published:** 2017-10-31

**Authors:** Francisco J. Ibáñez, Mónica A. Farías, Angello Retamal-Díaz, Janyra A. Espinoza, Alexis M. Kalergis, Pablo A. González

**Affiliations:** ^1^Departamento de Genética Molecular y Microbiología, Facultad de Ciencias Biológicas, Millennium Institute on Immunology and Immunotherapy, Pontificia Universidad Católica de Chile, Santiago, Chile; ^2^Departamento de Endocrinología, Escuela de Medicina, Facultad de Medicina, Pontificia Universidad Católica de Chile, Santiago, Chile; ^3^Institut National de la Santé et de la Recherche Médicale U1064, Nantes, France

**Keywords:** heme oxygenase-1, carbon monoxide, pharmacological induction, antiviral drug, herpes simplex virus, capsid

## Abstract

Heme oxygenase-1 (HO-1) is an inducible enzyme that is expressed in response to physical and chemical stresses, such as ultraviolet radiation, hyperthermia, hypoxia, reactive oxygen species (ROS), as well as cytokines, among others. Its activity can be positively modulated by cobalt protoporphyrin (CoPP) and negatively by tin protoporphirin (SnPP). Once induced, HO-1 degrades iron-containing heme into ferrous iron (Fe^2+^), carbon monoxide (CO) and biliverdin. Importantly, numerous products of HO-1 are cytoprotective with anti-apoptotic, anti-oxidant, anti-inflammatory, and anti-cancer effects. The products of HO-1 also display antiviral properties against several viruses, such as the human immunodeficiency virus (HIV), influenza, hepatitis B, hepatitis C, and Ebola virus. Here, we sought to assess the effect of modulating HO-1 activity over herpes simplex virus type 2 (HSV-2) infection in epithelial cells and neurons. There are no vaccines against HSV-2 and treatment options are scarce in the immunosuppressed, in which drug-resistant variants emerge. By using HSV strains that encode structural and non-structural forms of the green fluorescent protein (GFP), we found that pharmacological induction of HO-1 activity with CoPP significantly decreases virus plaque formation and the expression of virus-encoded genes in epithelial cells as determined by flow cytometry and western blot assays. CoPP treatment did not affect virus binding to the cell surface or entry into the cytoplasm, but rather downstream events in the virus infection cycle. Furthermore, we observed that treating cells with a CO-releasing molecule (CORM-2) recapitulated some of the anti-HSV effects elicited by CoPP. Taken together, these findings indicate that HO-1 activity interferes with the replication cycle of HSV and that its antiviral effects can be recapitulated by CO.

## Introduction

Heme oxygenase-1 (HO-1), also known as heat shock protein 32 (Hsp32) in mammals, is an inducible enzyme expressed in numerous cell types in response to increasing amounts of its substrate heme, as well as various stress stimuli, such as reactive oxygen species (ROS; Keyse and Tyrrell, 1989), ultraviolet radiation (Keyse and Tyrrell, 1987), hyperthermia (Shibahara et al., 1987), hypoxia (Murphy et al., 1991), as well as cytokines (Terry et al., 1998), and lipopolysaccharide (LPS; Camhi et al., 1995), among others (Ryter et al., 2006). Additionally, the activity of HO-1 can be modulated by numerous chemical compounds, such as protoporphirins either to increase or inhibit its activity. While cobalt protoporphyrin (CoPP) is known to promote the expression and activity of HO-1 (Bonkovsky et al., 1990; Shan et al., 2000), tin protoporphirin (SnPP) blocks its activity, although it may also elicit increased HO-1 expression (Bonkovsky et al., 1990; Ewing et al., 2005). Once induced, HO-1 has been described to localize at multiple sites within the cell, such as the endoplasmatic reticulum (Yoshida et al., 1988), plasma membrane (Kim et al., 2004), mitochondria (Slebos et al., 2007; Riquelme et al., 2015b), and nucleus (Lin et al., 2007), with its distribution likely playing different and specific roles at distinct organelles (Kim et al., 2004). HO-1 degrades iron-containing pro-oxidant heme (preferably heme *b* and *c* and hematoheme) into equimolar quantities of ferrous iron (Fe^2+^), carbon monoxide (CO) and biliverdin, with the latter rapidly being converted into bilirubin by NADPH-dependent biliverdin reductase (Tenhunen et al., 1970; Maines and Kappas, 1977). Importantly, numerous products of HO-1 catalysis are cytoprotective displaying anti-apoptotic, anti-oxidant, anti-inflammatory, and anti-cancer effects, among others (Ryter et al., 2006). Ferrous iron ions derived from HO-1 can participate in important cell processes that depend on this metal and high concentrations can modulate the stability of particular mRNAs, either dampening or promoting their translation (Eisenstein et al., 1991). Increased intracellular levels of iron also promote the expression of ferritin, which has been described to exert, *per-se* numerous cytoprotective effects against cell-damaging agents (Vile et al., 1994). Eventually, elevated cellular concentrations of iron derived from HO-1 activity may also activate cytoprotective NF-κB pathways, that support resistance to Fas-mediated apoptosis (Choi et al., 2004). While biliverdin and bilirubin display cytoprotective effects and act as strong anti-oxidants, at high concentrations they can alter mitochondrial function and be toxic for organs such as the brain (Menken et al., 1966; Clark et al., 2000; Kapitulnik, 2004). Lastly, carbon monoxide (CO) has acquired considerable attention as a molecule that modulates inflammatory processes, as well as cellular apoptosis (Motterlini and Otterbein, 2010). Among others, CO has been shown to dampen the expression of pro-inflammatory molecules on the cell surface (Riquelme et al., 2015a), alter endosome-lysosome fusion (Tardif et al., 2013), modulate mitochondrial function (Riquelme et al., 2015b), inhibit T cell activation (Mackern-Oberti et al., 2015) and alter cellular ion channel function (Peers et al., 2015).

Because cell infection with viruses often involves the modulation of stress-related processes that can favor or dampen virus replication, assessing the potential role of HO-1 over virus infection has acquired significant interest in the recent years. Indeed, new studies have shown that HO-1 displays important antiviral properties against human viruses, such as the human immunodeficiency virus (HIV; Devadas and Dhawan, 2006), influenza (Hashiba et al., 2001), human respiratory syncytial virus (RSV, Espinoza et al., 2017), hepatitis B (HBV; Protzer et al., 2007), hepatitis C virus (HCV; Lehmann et al., 2010), enterovirus 71 (EV71; Tung et al., 2011), dengue virus (DENV; Olagnier et al., 2014; Tseng et al., 2016), and Ebola virus (EBOV; Hill-Batorski et al., 2013). An antiviral role for HO-1 has also been reported for bovine viral diarrhoa virus (BVD; Zhang et al., 2015) and porcine reproductive and respiratory syndrome virus (PRRSV; Xiao et al., 2014) *in vitro*. While the mechanisms underlying the antiviral effects of HO-1 have remained elusive for some viruses, in other cases its antiviral effects have been identified (Schmidt et al., 2012). For instance, bilirubin derived from HO-1 has been reported to inhibit the protease activity of HIV (Liu et al., 2016), the activity of the non-structural 3/4A protease of HCV (Zhu et al., 2010) and non-competitively inhibit the protease of DENV (Olagnier et al., 2014). Interestingly, biliverdin derived from HO-1 has also been shown to elicit an increased interferon response against HCV (Lehmann et al., 2010). On the other hand, iron derived from HO-1 has been suggested to inhibit the non-structural 5B (NS5B) RNA-dependent RNA polymerase of HCV, through the inhibition of divalent cation binding (Fillebeen et al., 2005). Finally, CO has been shown to dampen ROS generation in EV71-infected cells and consequently diminish virus replication in these cells (Tung et al., 2011).

Given that the products of HO-1 can exert significant anti-viral effects, beyond their anti-oxidant and cytoprotective properties, studying the role of this enzyme over the replication of viruses may help identify novel therapeutic approaches that hamper viral infection. Herpes simplex viruses (HSV-1 and HSV-2) are highly prevalent in the human population with scarce treatment options for drug-resistant variants that may arise in immunosuppressed individuals and at present, there are no available vaccines to prevent infection (Suazo et al., 2015). While HSV-1 is the primary cause of infectious blindness in developed countries, HSV-2 is a major catalyst of HIV infection and spread (Suazo et al., 2015). Building on a previous study that reported that bilirubin, a product of heme metabolism by HO-1 can dampen HSV replication *in vitro* (Santangelo et al., 2012), we sought to assess the role of HO-1 activity on the infection of epithelial and neuronal cells, to identify other potential antiviral products of this enzyme and assess possible mechanisms of action. By using HSV viruses that encode structural and non-structural green fluorescent protein (GFP) genes, here we assessed the effects of the pharmacological induction of HO-1 activity over cell infection with HSV-2 and found that treatment with CoPP impaired virus propagation. Furthermore, CoPP treatment protected epithelial cells from suffering morphological cytopathology after infection. Interestingly, many effects elicited by CoPP were recapitulated by CO. Taken together, these results propose an important role for HO-1 and its product CO in blocking HSV infection thus, opening potential new treatment opportunities against this virus. Noteworthy, numerous CO-releasing molecules (CORMs) intended for clinical use are currently being developed (Zobi, 2013; Abeyrathna et al., 2017).

## Materials and methods

### Cells and viruses

Vero (ATCC #CCL-81) cells were used to propagate HSV-2 (strain G; Dolan et al., 1998). Briefly, T75 flasks with Vero cell monolayers were grown in RPMI (Thermo Fisher Scientific), 5% FBS (Fetal Bovine Serum Gibco®, Thermo Fisher Scientific) supplemented with 1 mM piruvate (Thermo Fisher Scientific), 2 mM Glutamine (Thermo Fisher Scientific) and 100 IU/mL Penicilin/Streptomycin (Thermo Fisher Scientific) to 80% confluence, inoculated with an MOI of 0.01 of virus in 10 ml Opti-MEM media (Thermo Fisher Scientific) and incubated at 37°C for 1 h. Then, supernatants were replaced with fresh Opti-MEM medium for 24–36 h until abundant visible cytopathic effect was observed. The contents of the flasks were pooled, and cells removed twice by centrifugation at 400 g for 10 min. Pellets were sonicated for 5 min in a sonicator waterbath (Branson 1210, Branson Ultrasonics), aliquoted in cryotubes and stored at −80°C until use. Virus dilutions were titered over Vero cells cultured in flat-bottom 96 well plates and screened for plaque formation after cell fixation with 1% paraformaldehyde (PFA, Winkler) in PBS and a 0.04% crystal violet (Sigma-Aldrich) staining solution.

### Modulation of HO-1 activity and CORM-2

Vero and HeLa cells (ATCC, #CCL-2) were grown in RPMI supplemented as indicated above, to 80% confluence before application of the treatments indicated below. Vero cells were treated with 60 μM CoPP (Frontier Scientific, Inc.), 60 μM SnPP (Frontier Scientific, Inc.) or an equivalent volume (2.24 μL per mL) of NaOH 0.1 M (vehicle for CoPP and SnPP) in Opti-MEM (Thermo Fisher Scientific) for 6 h, then the cells were washed with PBS. HeLa cells were treated with 10 μM CoPP, 10 μM SnPP, or an equivalent volume of NaOH 0.1 M (vehicle for CoPP and SnPP) in Opti-MEM for 14 h, then washed with PBS. Next, Vero or HeLa cells were inoculated with HSV-2 at the indicated MOI for 1 h in Opti-MEM media at 37°C, then washed with PBS and finally incubated in Opti-MEM for additional periods, as indicated for each experiment, and prepared for western blot analysis, flow cytometry, plaque formation, or laser confocal microscopy. Tricarbonyldichlororuthenium(II) dimer, also known as CORM-2 (Sigma Aldrich, catalog number #288144), was used as a carbon monoxide donor. CORM-2 was applied to cells 6 h after infection with HSV at a final concentration of 100 μM in Opti-MEM medium, as previously reported by others (Zhang et al., 2017). Lyophilized CORM-2 was pre-dissolved DMSO at a final concentration of 100 mM and then diluted as needed in Optimem before application onto the cells. DMSO was used as a vehicle when assessing the effect of CORM-2. CORM-2 was inactivated (iCORM-2) with 0.1 M HCl for 2 h and then the neutralized with 0.1 M NaOH. As a control, CORM-2 was diluted with equivalent amounts of NaCl at neutral pH.

### Western blot analyses

Western blot analyses were performed to assess the expression of HO-1 and viral proteins. Briefly, protein preparations from 0.4 × 10^6^ cells were extracted using RIPA protein extraction RIPA buffer (2006). Proteins in the soluble fraction were then quantified using Pierce BCA Protein Assay Kit (Thermo Fisher Scientific). Twenty-five micrograms of protein was loaded onto SDS-PAGE 12% polyacrylamide gels (miniprotean II, Bio-Rad Laboratories) and transferred onto nitrocellulose membranes (Promega). After transfer, membranes were blocked with 5% Bovine Serum Albumin (BSA, Winkler, Chile) and incubated, either with an anti-HO-1 monoclonal antibody (Abcam, clone ab13248) at a dilution of 1:400 at 4°C, an anti-gD monoclonal antibody (Virusys, clone HA025) at a dilution of 1:50,000 at 4°C, an anti-VP16 monoclonal antibody (Santa Cruz Biotechnology, clone 1-21) at a dilution of 1:1000 at 4°C overnight or an anti-β-actin (Biolegend, clone 2F1-1) at a dilution of 1:1,500 for 2 h at room temperature. After incubation, membranes were washed thrice with TBS-Tween 0.01% (Calbiochem, Inc. La Jolla) and incubated with an anti-mouse-IgG HRP-conjugated polyclonal antibody (Biolegend, Poly4053) for 1 h at room temperature at a dilution of 1:2,500. After incubation with the secondary antibody, membranes were washed, membranes were washed thrice with TBS-0.01% Tween and incubated with a luminol:coumaric acid solution to detect membrane-bound antibodies. Quimioluminiscence derived from this reaction was visualized using a MyECL™ Imager (Thermo Fisher scientific) digital documentation system. Band intensity was calculated using UN-SCAN-IT gel 6.1 software (Silk Scientific Corporation).

### Quantitative PCR (qPCR)

Vero and HeLa cells were left untreated or treated with HO-1-modulating drugs at 60 and 10 μM, respectively and then infected with HSV-2 G strain at an MOI 10. Samples were collected at the indicated time-points after infection and processed for DNA extraction. Briefly, cells and cell supernatants were ultracentrifugated at 21,000 × g to pellet both, cells and virus particles in the supernatants. The pellet was then processed according to the method described for Extraction and Precipitation of DNA in Appendix 3C of the Current Protocols Humman Genetics 2001. The DNA was then used for qPCR analysis using 100 ng of DNA per reaction with the following primers and probe for the *U*_*L*_*30* gene: Fwd GGCCAGGCGCTTGTTGGTGTA, Rev-ATCACCGACCCGGAGAGGGA and Probe CCGCCGAACTGAGCAGACACCCGC and an Applied Biosystems StepOnePlus thermocycler, as previously described (Petro et al., 2016).

### Flow cytometry

To evaluate HO-1 expression by flow cytometry, 0.5–1.0 × 10^6^ cells were detached using 0.25% W/V Trypsin (Thermo Fisher Scientific) for 10 min at 37°C, centrifugated at 400 g, washed with PBS and fixed for 15 min at room temperature with 4% paraformaldehyde (PFA). Then cells were permeabilized with 0.05% saponin in PBS (Sigma-Aldrich, St Louis USA) for 45 min at 4°C, washed with PBS and incubated with an anti-HO-1 antibody (Abcam, clone ab13248) at a dilution of 1:400 in PBS-saponin 0.1% for 1 h. Cells were then washed twice with PBS and subsequently incubated with an anti-mouse-IgG-APC antibody (Biolegend, clone Poly4053) for 45 min at 4°C. Finally, cells were washed twice with PBS and resuspended in PBS for flow cytometry analysis. To evaluate GFP-derived fluorescence from HSV GFP-capsids (structural reporter) or the non-structural GFP reporter encoded within the virus genome, 0.5 × 10^6^ cells Vero or HeLa were infected with the indicated amounts of virus for 1 h in Opti-MEM, washed with PBS and then incubated with Opti-MEM for an additional 16 h prior to trypsinization and fixation as indicated above, but for 20 min. Cell viability was assessed by flow cytometry using the Zombie-NIR Fixable Viability Kit (BioLegend). All flow cytometry analyses were performed on a FACSCANTO II flow cytometer (BD Beckton Dickinson).

### Multi-mode plate reader

GFP-derived fluorescence from HSV GFP-capsids or GFP encoded within the virus genome was assessed within SH-SY5Y cells treated with CORM-2 or inactivated CORM2 (iCORM2) on a Synergy Neo HTS Multi-Mode Reader (Biotek Instruments, Inc.).

### Laser confocal microscopy

1 × 10^4^ Vero or HeLa cells were seeded onto Slide 8-well FLux Hybridwell™ microchambers (SPL Life Sciences Co., Korea) for 16 h at 37°C in RPMI, then cooled to 4°C for 5 min and incubated with HSV VP26-GFP at an MOI of 400 for 1 h to 4°C. Later, cells were transferred to a culture chamber at 37°C to synchronize virus internalization. Two hours later, cells were washed twice with PBS and fixed with 2% PFA at 4°C for 20 min. Then the cells were washed twice and stained with Hoechst (Thermofisher Scientific, #H21492) in PBS at a final concentration of 2 μg/ml for 15 min. Then, the cells were washed twice and stained 10 min with Alexa Fluor 594 wheat germ agglutinin, (WGA, Thermofisher Scientific, #W11262) in PBS at final concentration of 5 μg/ml. Finally, the cells were washed twice and mounted with Prolong Diamond Antifade Mountant (Thermofisher Scientific, #P36970). Microscopic analyses were performed at 63x in a Ti Eclipse, Nikon laser confocal microscope. An average of 15 fields and 150–200 cells were analyzed per experiment. The distribution of capsid-associated fluorescence between treatments was assessed in a blinded manner. To determine capsid-derived fluorescence in the cytoplasm of infected cells, the images obtained by confocal microscopy were analyzed with Image J and the FIJI plugin, according to a report by McCloy et al. (2014). Briefly, both regions of interest consisting on cells, based on WGA-staining (cell membrane label), as well as regions free of cells, to determine background fluorescence were selected. The selected regions were then analyzed in the green channel (GFP) and converted to an 8 bit format. Then, the area and the integrated fluorescence density of each region of interest (cell) were calculated in z-stack images. Finally, the value of the TCCF in the complete z-axis of the cell was calculated. TCCF is the integral density of fluorescence of the region of interest minus the equivalent of the cell area^*^mean fluorescence intensity of the background.

### Statistical analysis

Statistical significance between experimental groups was assessed by unpaired Student's *t*-test (bar graphs), one-way analysis of variance (ANOVA) with Bonferroni's multiple comparison test (three or more groups) or two-way analysis of variance with Tukey's multiple comparison test (two independent variables) using GraphPad Prism (GraphPad Software, La Jolla California USA). Values were considered statistically significant for *p* ≤ 0.05.

## Results

### HO-1 expression in CoPP-treated and HSV-infected epithelial cells

**Figure 1:**
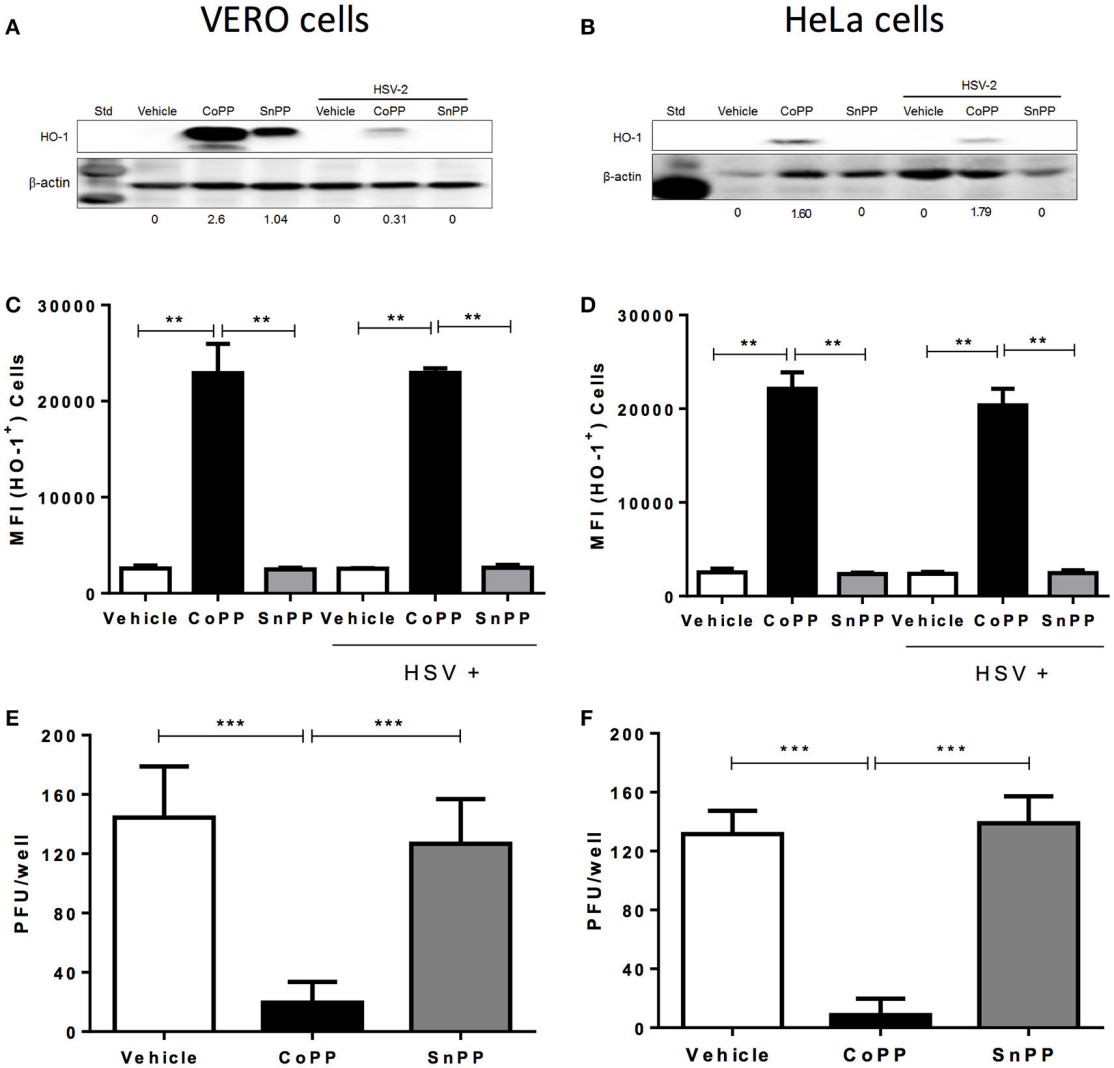
HO-1 expression in epithelial cells treated with CoPP or SnPP and infected with HSV. **(A,B)** Western blot analyses of HO-1 expression in Vero (left) and HeLa cells (right) after treatment with HO-1-modulating drugs and/or infection with HSV-2 at an MOI 1 for 16 h. **(C,D)** Flow cytometry analyses of HO-1 expression in Vero (left) and HeLa cells (right) after treatment with HO-1-modulating drugs and/or infection with HSV-2 at an MOI 1 (MFI, mean fluorescence intensity). **(E,F)** Virus plaque formation (PFU, plaque forming units) in Vero (left) and HeLa cells (right) treated with HO-1-modulating drugs and then infected with 150 PFU of HSV-2. Plaque forming units were determined at 18 and 36 h post-infection for Vero and HeLa cells, respectively. Data are means ± SEM of three independent experiments. Representative images are shown for western blots. Two-way ANOVA, and Tukey's multiple comparison test were used for statistical analyses (^**^p < 0.01, ^***^p < 0.001).

To determine whether HSV modulates the expression of HO-1 in cells that are permissive for infection with this virus, we assessed the expression of HO-1 by western blot and flow cytometry in Vero and HeLa cells treated with CoPP or SnPP. Vero cells are monkey kidney epithelial cells (*Cercopithecus aethiops;* green monkey), frequently used for expanding viruses, while HeLa is a human cervix epithelial cell line that is susceptible to herpes simplex virus infection. As shown in Figures 1A,B, Vero cells and HeLa cells, respectively up-regulated the expression of HO-1 in response to CoPP. The concentration of CoPP that induced optimal HO-1 expression, altogether without compromising cell viability was both, based on previous reports and determined experimentally by performing dose-response curves (Supplementary Figure 1; Zhang et al., 2015; Espinoza et al., 2017). Overall, the expression of HO-1 did not significantly vary upon HSV-2 infection at an MOI 1 in the absence of treatment in these cells as compared to non-infected controls. Other MOIs tested and incubation periods with the virus did not significantly alter the expression of HO-1 in these cells (data not shown). Nevertheless, Vero cells treated with CoPP evidenced a significant increase in the expression of HO-1, which decreased after HSV-2 infection when assessed by western blot; however, this was not the case for HeLa cells. Treatment with SnPP, a drug that inhibits the activity of HO-1 (Sardana and Kappas, 1987), yet may up-regulate its expression in some cells (Ewing et al., 2005), increased the expression of this enzyme in Vero cells without infection (western blot). HeLa cells treated with SnPP did not express HO-1 with or without infection, suggesting that the modulation of HO-1 expression by SnPP is likely restricted to certain cells types and/or culture conditions (Figure 1A). Flow cytometry analyses of HO-1 expression in these cells overall revealed similar results to those observed by western blot, that is no variation in HO-1 expression after HSV-2 infection and significant HO-1 expression after CoPP treatment (Figures 1C,D). Similar results were also observed in SH-SY5Y cells, which are human neuronal cells derived from the SK-N-SH neuroblastoma cell line (Supplementary Figures 2A,B).

To evaluate whether increased expression of HO-1, induced by CoPP modulates the replication cycle of HSV-2 within permissive cells, we infected Vero and HeLa cells with a fixed amount of virus (150 PFU) and measured virus plaque formation. As shown in Figures 1E,F, Vero and HeLa cells treated with CoPP and infected with HSV-2 displayed significantly less plaque forming units (PFU) at 18 and 36 h after infection, as compared to untreated cells or cells treated with SnPP. Similar results were observed with the SH-SY5Y neuronal cell line, which released significantly less infectious HSV particles at 24 h post-infection when treated with CoPP (Supplementary Figure 2C).

Taken together, these results suggest that HSV does not modulate HO-1 expression *per-se* in infected cells, nor significantly interferes with its expression after pharmacological induction with CoPP. Furthermore, we observe that CoPP treatment reduces virus plaque formation in Vero and HeLa cells, while SnPP does not, suggesting that the effect of HO-1 over HSV replication depends on its catalytic activity.

### CoPP restricts HSV-encoded gene expression in infected cells

**Figure 2:**
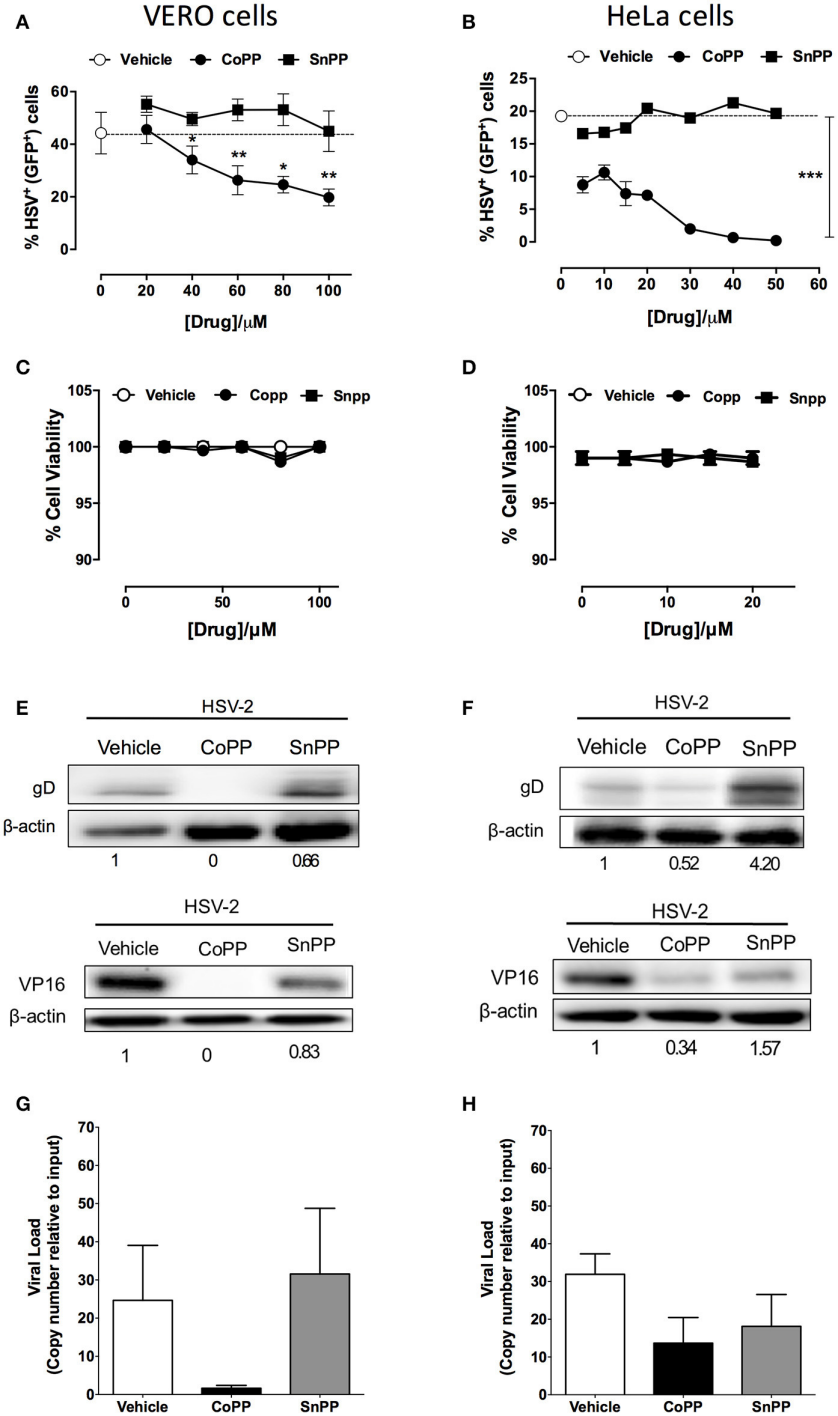
Pharmacological induction of HO-1 restricts the expression of HSV-encoded genes in epithelial cells. **(A,B)** Flow cytometry analyses of GFP-derived fluorescence in Vero (left) and HeLa cells (right), respectively infected at MOI 1 with HSV-2 that encodes a non-structural GFP reporter gene under the control of a constitutive promotor, in response to varying concentrations of HO-1-modulating drugs. **(C,D)** Cell viability was assessed for Vero (left) and HeLa cells (right) for treatment with varying concentrations of HO-1-modulating drugs. **(E,F)** Western blot analyses for viral proteins gD and VP16 in Vero (left) and HeLa (right) cells at 16 h post-infection with HSV-2 at an MOI 1 after treatment with 60 and 10 μM, respectively of HO-1-modulating drugs. **(G,H)** Quantification of viral genome copies in Vero (left) and HeLa cells (right) by qPCR at 18 h post-infection at MOI 10. Data are means ± SEM of three independent experiments for experiments **(A–F)** and two independent experiments for **(G,H)**. Representative images are shown for Western blots. Two-way ANOVA, and Tukey's multiple comparison test were used for statistical analyses **(A)**: statistics is shown for the CoPP treated group vs. SnPP-treated and untreated (^*^p < 0.05, ^**^p < 0.01, ^***^p < 0.001).

To determine whether reduced virus plaque formation in cells treated with CoPP was due to reduced viral protein expression within inoculated cells, we assessed the expression of both, a reporter gene encoded within the virus genome, as well as viral genes in CoPP-, SnPP-treated and untreated cells. The reporter gene used in this assay consists of a non-structural GFP controlled by a constitutive, HSV-independent strong promoter (human cytomegalovirus, CMV; Wang et al., 2012). Noteworthy, Vero cells (Figure 2A) and HeLa cells (Figure 2B) treated with increasing concentrations of CoPP and then inoculated with HSV at an MOI 1, expressed significantly less GFP than cells treated with vehicle alone or SnPP. A similar result was observed for SH-SY5Y cells treated with these drugs and inoculated with the virus (Supplementary Figures 3A,B). To exclude the possibility that these results may be a consequence of increased cell death after treatment with CoPP or SnPP, we assessed cell viability for all treatments. Importantly, neither CoPP nor SnPP treatment significantly altered the viability of Vero, HeLa, or SH-SY5Y cells at the drug concentration used (Figures 2C,D and Supplementary Figure 3C, respectively).

To determine whether reduced expression of the virus-encoded GFP protein also applied to virus-encoded viral proteins, we performed western blot assays for HSV glycoprotein D (gD) and VP16 in cells treated or not with vehicle, CoPP and SnPP. As shown in Figure 2E the expression of gD and VP16 was significantly reduced in Vero cells treated with CoPP, as compared to untreated cells and cells treated with SnPP. However, the latter also displayed a slight reduction in viral protein expression upon treatment. Similar results were observed for HeLa cells, although treatment with SnPP increased viral protein expression (Figure 2F). Equivalent findings to HeLa cells were observed for SH-SY5Y cells (Supplementary Figure 3D), suggesting that pharmacological induction of HO-1 with CoPP in epithelial and neuronal cells interferes with the expression of HSV-encoded proteins. To assess whether CoPP treatment also affected the quantity of viral genomes present in Vero and HeLa cells, we performed qPCR for the *U*_*L*_*30* viral gene on total DNA obtained from each treatment. As shown in Figures 2G,H, CoPP-treatment reduced the amount of HSV genome copies recovered at 16 h after infection of both, Vero and HeLa cells with an MOI 10, although the differences were less marked in HeLa cells and in both cases did not reach statistical significance.

### CoPP treatment does not interfere with virus binding or entry

**Figure 3:**
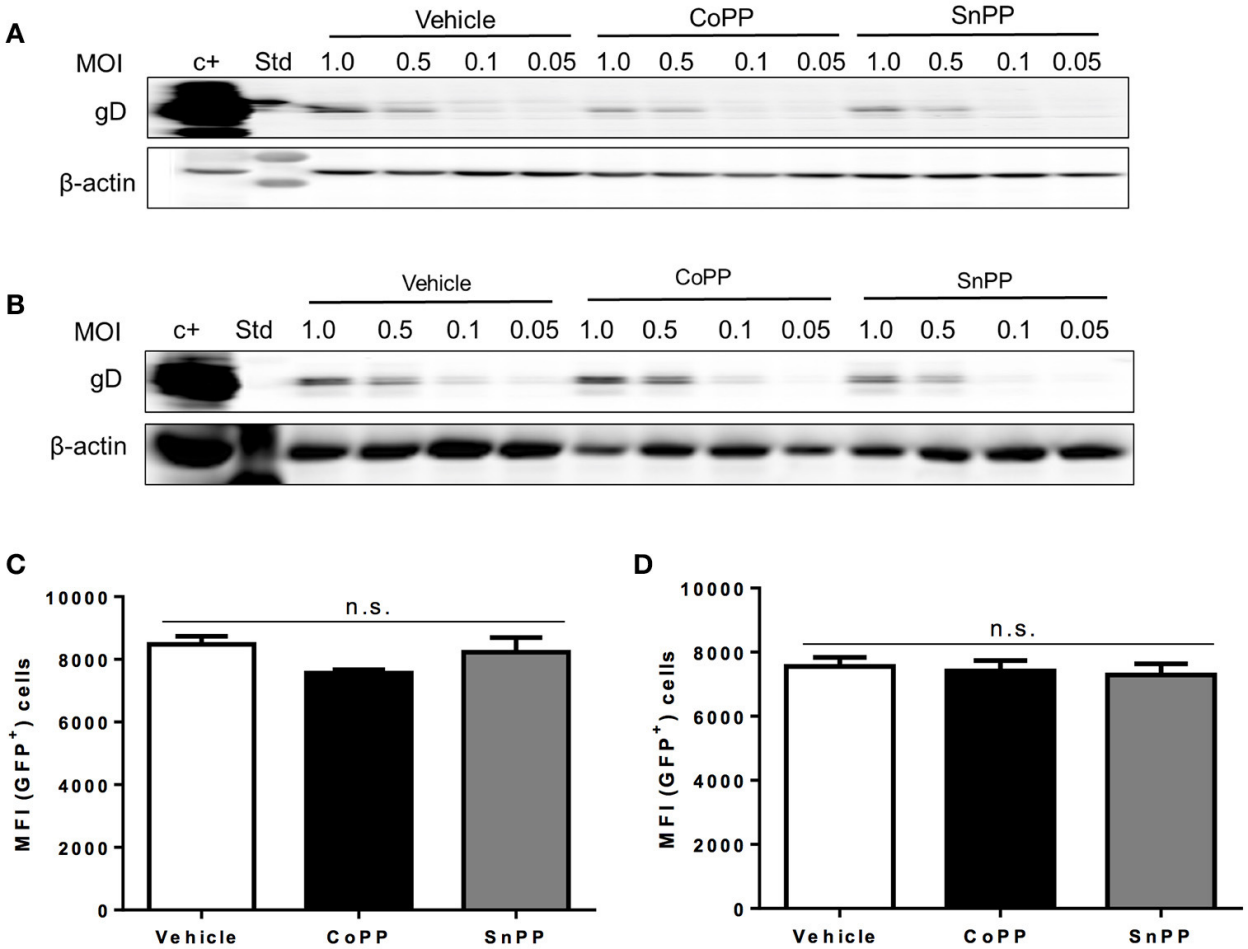
Pharmacological induction of HO-1 activity does not interfere with virus binding to the cell surface nor capsid entry into the cytoplasm. Western blot analyses for determining virus binding to the surface of **(A)**. Vero cells and **(B)**. HeLa cells. Cells were incubated with varying doses of virus for 5 h at 4°C, washed with saline buffer and then immediately prepared for protein extraction. HSV gD protein expression was assessed to determine the amount of virus bound to the surface of cells. **(C,D)** Flow cytometry analyses measuring virus-derived GFP fluorescence of capsids internalized into the cytoplasm 2 h post-infection with a MOI of 100 after treatment with CoPP, SnPP, or vehicle in Vero cells (left) and HeLa cells (right). Cells were thoroughly washed with saline buffer and treated with trypsin to remove any surface-bound virus before analysis. Data are means ± SEM of three independent experiments. Representative images are shown for Western blots. Two-way ANOVA, and Tukey's multiple comparison test were used for statistical analyses (n.s., non-significant differences).

To gain insights on the mechanism of action of HO-1 activity over impaired HSV replication and viral protein expression in CoPP-treated cells, first we assessed whether equivalent amounts of virus bound to the surface of drug-treated and untreated epithelial cells, in such a way to determine whether reduced virus binding to the cell surface may account for lesser infection and hence, diminished virus gene expression. To evaluate virus binding to the cell surface, we performed a previously described virus-binding assay that assesses attachment of different amounts of virus to the cell surface at 4°C (Cheshenko et al., 2013). As shown in Figures 3A,B, western blot analyses of gD evidenced equivalent amounts of virus bound to the surface of untreated, CoPP- and SnPP-treated Vero and HeLa, respectively suggesting that pharmacological treatment with these drugs does not interfere with the expression of virus-binding proteins on the surface of epithelial cells, nor blocks virus binding to these cells.

To further dissect how the pharmacological induction of HO-1 with CoPP interferes with HSV replication, we used an HSV virus that encodes a GFP-VP26 fusion protein (structural reporter) and measured capsid-derived GFP fluorescence early after infection to evaluate capsid entry into the cytoplasm of treated and untreated cells. To minimize the detection of GFP fluorescence that may derive from virus particles bound to the cell surface, that have not been internalized into the cytoplasm, cells were washed and treated with trypsin before flow cytometry analyses. As shown in Figure 3, equivalent amounts of capsid-derived GFP fluorescence was detectable intracellularly for all treatments in Vero (Figure 3C) and HeLa cells (Figure 3D), indicating that similar quantities of virus internalize in treated and untreated cells. These results suggest that CoPP-treatment does not interfere with the capacity of the virus to enter target cells and thus, that interference with gene expression likely occurs further downstream of this process.

### Viral capsid distribution in CoPP-treated infected cells

**Figure 4:**
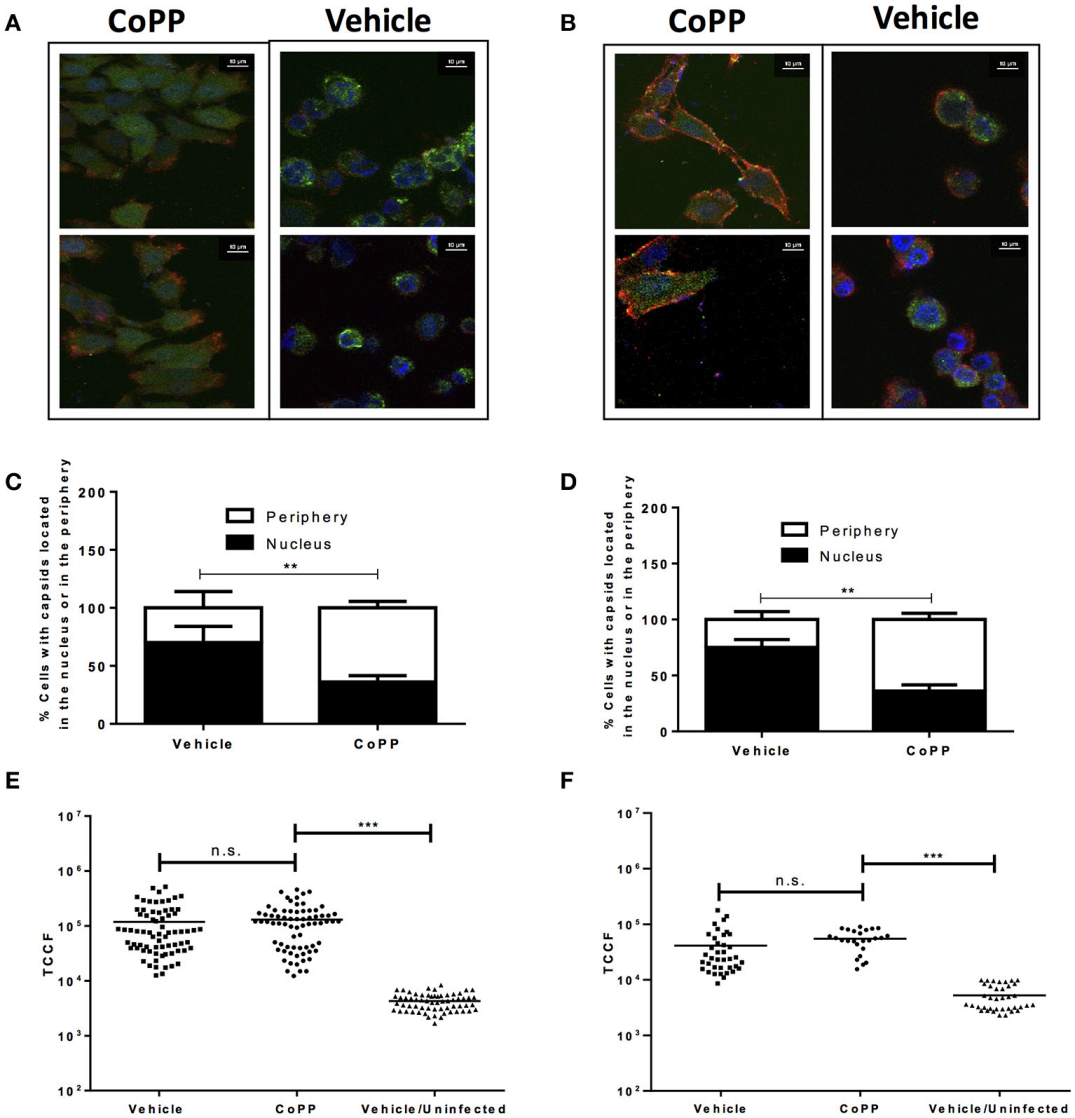
Pharmacological induction of HO-1 with CoPP modulates capsid distribution in infected cells and elicits differential cell morphology after treatment after HSV-infection. Representative confocal microscopy images of **(A)**. Vero and **(B)**. HeLa cells treated or not with CoPP and infected with an HSV virus encoding a structural GFP fluorescent capsid (green: GFP-capsid fusion protein). Cell nuclei were stained with Hoescht (blue), and membrane were stained with WGA (red) **(C,D)**. Quantification of the distribution of capsid GFP-fluorescence in treated and infected Vero (left) and HeLa cells (right), respectively relative to the position of the nucleus. An average of fifteen fields and 150–200 cells were analyzed per experiment in a blind manner **(E,F)**. Quantification of total green fluorescence (virus encoded structural GFP fluorescent capsid) in confocal microscopy images of HSV-infected Vero (left) and HeLa cells (right), respectively treated or not with CoPP. TCCF is the integral density of fluorescence of the region of interest minus the equivalent of the cell area^*^mean fluorescence intensity of the background in the complete z-axis of the analyzed cells. Representative confocal microscopy images at 63X magnification are shown. Data are means ± SEM of two independent experiments. One way ANOVA, Two-way ANOVA, and Tukey's multiple comparison test were used for statistical analyses (^**^p < 0.01, ^***^p < 0.001).

The following process associated to the virus infectious cycle that we assessed was capsid accumulation at the outer nuclear membrane in virus-inoculated cells. Upon infection, HSV capsids entering the cytoplasm migrate from the inner side of the plasma membrane to the nucleus to deliver the viral genome into this compartment. To evaluate whether CoPP-treatment interferes with the accumulation of viral capsids around the nucleus, we performed confocal microscopy analyses on cells inoculated with a virus containing GFP-fluorescent capsids 2 h after viral entry was allowed. Importantly, we observed that CoPP-treated Vero cells displayed less capsid-derived GFP fluorescence distributed adjacent to the nucleus, as compared to non-treated cells and that the viral capsids in CoPP-treated cells were rather homogenously dispersed in the cytoplasm (Figures 4A,C). A similar result was observed for CoPP-treated HeLa cells (Figures 4B,D). Because the intensity of the virus-derived fluorescence seemed somewhat lower in cells treated with CoPP, as compared to vehicle-treated cells, we quantified the total intensity of this fluorescence in individual cells using z-stack confocal imaging. As shown in Figures 4E,F, the integral density of fluorescence of individual Vero and HeLa cells that were infected with HSV was calculated in three dimensions and found not to be significantly different between treatments. This result indicates that similar amounts of virus entered the cells, which is consistent with the flow cytometry data in Figures 3C,D. However, it is noteworthy to mention that vehicle-treated epithelial cells displayed a sphere-shaped phenotype after HSV infection which was not observed in CoPP-treated cells. Indeed, cells treated with CoPP retained their epithelial-like phenotype after drug treatment and HSV infection which may impact the distribution and concentration of viral capsids within these cells and negatively affect the virus replication cycle (Figures 4E,F).

### Carbon monoxide recapitulates the effects of CoPP in epithelial cells

**Figure 5:**
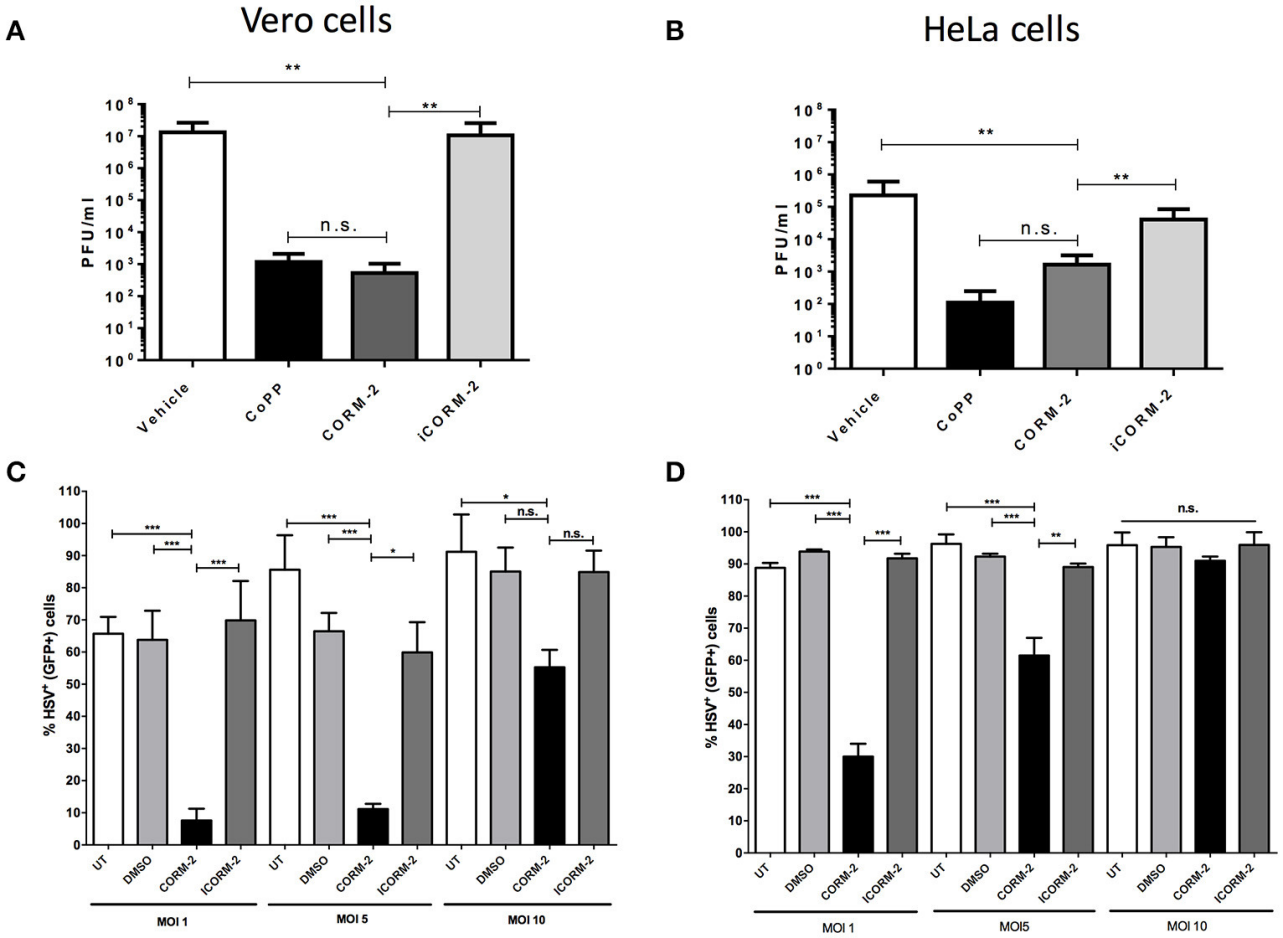
Treatment with a carbon monoxide-releasing molecule recapitulates the antiviral effects of CoPP after HSV infection. Quantification of plaque forming units (PFU) after HSV-2 titration over **(A)**. Vero and **(B)**. HeLa cells treated with CORM-2 and inactivated CORM-2 (iCORM2). Virus-encoded GFP fluorescence (non-structural GFP reporter) determined by flow cytometry in **(C)**. Vero and **(D)**. HeLa cells infected with HSV at different MOIs at 20 h post-infection. Data are means ± SEM of three independent experiments. One-way ANOVA, and Tukey's multiple comparison test were used for statistical analyses (^*^p < 0.05, ^**^p < 0.01, ^***^p < 0.001).

Because HO-1 yields three enzymatic products as a consequence of its activity, we sought to assess whether one of its products, namely carbon monoxide (CO), which is known to modulate the expression of pro-inflammatory molecules by the cell (Riquelme et al., 2015a), alter endosome-lysosome fusion (Tardif et al., 2013) and modulate mitochondrial function (Riquelme et al., 2015b), among others plays a role in the results observed above. As shown in Figures 5A,B, HSV titered out at significantly higher virus dilutions in CORM-2-treated Vero and HeLa cells, respectively than untreated cells. The effect conferred by CORM-2 treatment was similar to that observed for CoPP. Importantly, inactivation of CORM-2 (iCORM-2) reestablished the titters obtained with vehicle alone. Furthermore, Vero and HeLa cells treated with CORM-2 displayed significantly less virus-related GFP fluorescence (non-structural reporter) than cells treated with vehicle alone (Figures 5C,D, respectively). Again, inactivation of CORM-2 (iCORM-2) yielded GFP- fluorescence levels similar to those obtained with the vehicle. A similar result was observed in SH-SY5Y cells, which displayed less virus-derived fluorescence after treatment with CORM-2 Supplementary Figure 4). Importantly, inactivation of CORM-2 (iCORM-2) restored the GFP fluorescence to similar levels as those observed in vehicle-treated cells. Taken together, these results suggest that the HO-1 product CO mediates numerous of the effects elicited by CoPP treatment.

## Discussion

Recent studies report significant antiviral properties for HO-1 and its enzymatic products. Here, we observed that pharmacological induction of HO-1 activity hampers HSV propagation in epithelial cells and neuronal cells. Furthermore, we found that carbon monoxide, a product of HO-1 activity could reproduce numerous of the effects of this enzyme.

A previous study that assessed the effect of bilirubin over HSV infection suggested that its inhibition over HSV replication may be mediated through the production of NO, which displays virucidal effects (Croen, 1993; Akaike and Maeda, 2000; Santangelo et al., 2012). Interestingly, CO can bind to target proteins that harbor heme groups, such as caveolar NO synthase (NOS) which promotes NO production, and modulate their functions by increasing for example their activity (Boczkowski et al., 2006). Interestingly, NO produced by NOS has been described to promote the activity of HO-1 leading to an activating positive loop between both gases (Zuckerbraun et al., 2003; Wegiel et al., 2010). Thereby, it is possible that the effect of CO observed herein might relate directly to the production of NO and relate to the previous results reported with bilirubin and HSV (Santangelo et al., 2012).

Additionally, CO displays numerous other molecular targets within cells with varying consequences, such as guanylyl cyclases (sGC), heme-containing channels, surface NADPH oxidase, and heme-containing transcription factors, such as NPAS2 (Dioum et al., 2002; Boczkowski et al., 2006). CO can also modulate the activity of mitochondrial proteins, such as mitochondrial cytochrome c oxidases (Desmard et al., 2007; Zuckerbraun et al., 2007) and promote mitochondria-derived ROS, although at very low levels, which have been described to act as signaling molecules (Almeida et al., 2015). Thus, it is likely that depending on the concentration of CO generated by HO-1 within the cell, that multiple functional outcomes by this molecule may arise.

Previous studies have also reported anti-microbial effects for CO, namely against several viruses. CO-mediated inhibition of EV71 virus replication in neuronal cells was suggested to occur through the inhibition of ROS levels that are deliberately induced by this virus in these cells (Tung et al., 2011). Distinct from the EV71 virus, HSV viruses are likely susceptible to pro-oxidizing environments, as they carry catalases in the virion that are intended to neutralize ROS in infected cells (Newcomb and Brown, 2012). Furthermore, inducing ROS in infected cells with trimeric and tetrameric derivatives of stilbenoids have been shown to inhibit HSV replication and thus, inhibition of ROS in target cells could rather favor these viruses (Chen et al., 2012).

While elevated levels of CO may dampen ATP production by the mitochondria and glycolysis separately, low levels of CO may also promote adequate levels of ATP production because of adaptive feedback loops triggered by mechanisms that sense hypoxic states in the cell (Lavitrano et al., 2004; Tsui et al., 2007). Importantly, reduced levels of ATP could impact cellular processes that require increased levels of this molecule, such as cargo transporters between cellular compartments (Dodding and Way, 2011; Kaczara et al., 2015; Riquelme et al., 2015b). Because HSV binds to ATP-consuming molecular motors, such as dynein within the cell, reduced levels of ATP could interfere at some level with the activity of this molecule, although the role of microtubules in capsid transport in epithelial cells is still a matter of discussion (Lee et al., 2006; Wolfstein et al., 2006; Abaitua et al., 2012; Matthews et al., 2013). Another process that requires elevated levels of ATP and is required for optimal HSV genome delivery into the nucleus is proteasome activity. Indeed, blocking energy-consumption by this protein complex has been described to inhibit optimal capsid migration within infected cells (Delboy et al., 2008). Hence, it is possible that altered availability of ATP within infected cells treated with CoPP or CORM-2, may compromise optimal HSV capsid accumulation around the nucleus, which is required early after infection for the initiation of an infectious replication cycle.

On the other hand, it is important to note that HO-1 and its product CO have been reported to directly interfere with the activity of host heat shock protein 90 (HSP90), which plays a crucial role in the localization of HSV capsids and their association with the nucleus (Lee et al., 2014; Zhong et al., 2014). Thus, it is also possible that CO could inhibit HSP90 activity, in such a way to interfere with the capacity of this protein to interact with HSV capsids and alter their localization within infected cells.

An interesting finding in this study was that while cells treated with CoPP retained their epithelial-like phenotypes after infection with HSV, vehicle-treated cells infected with this virus acquired a rounded-like phenotype (cytopathic-like phenotype). Importantly, the transition of an epithelial-like phenotype into a rounded cell morphology may better support viral capsid encounter with nuclear components that are needed for viral genome delivery into this compartment, as a role for microtubules in viral capsid transport to the nucleus remains controversial (Lee et al., 2006; Wolfstein et al., 2006; Abaitua et al., 2012; Matthews et al., 2013). Noteworthy, a recent study reported that the induction of HO-1 significantly increased the expression of adhesion molecules in cancer cells, namely E-cadherin and β-catenin, which can modulate the morphology of cells (Gueron et al., 2014).

Taken together, the results obtained in this study suggest an antiviral effect for HO-1 over HSV, which is at least partially mediated by its product CO. Although, our results point at the unusual distribution of viral capsids within infected cells as a possible mechanism by which HO-1 activity may interfere with the replication cycle of HSV, further experiments will be needed to determine whether this phenomenon overall accounts for reduced virus yield. Additionally, it will be of interest to determine if the effects elicited by HO-1 activity, and more specifically CO over HSV infection apply in the context of *in vivo* infections. Importantly, at present there are several CO-releasing molecules (CORMs) that are being tested in pre-clinical settings that replicate the effects of HO-1, when CO has been identified as a relevant effector of this enzyme (Motterlini and Otterbein, 2010; Schatzschneider, 2015).

## Author contributions

FI, MF, and PG designed experiments. FI, MF, and ARD conducted experiments. FI, MF, ARD, JE, AK, and PG analyzed the data. FI, AK, and PG wrote the manuscript. All authors reviewed the manuscript.

### Conflict of interest statement

The authors declare that the research was conducted in the absence of any commercial or financial relationships that could be construed as a potential conflict of interest.

## Supplementary material

Supplementary Figure 1 HO-1 expression in Vero and HeLa cells treated with low doses of CoPP. **(A,B)** Quantification of HO-1 expression in Vero (left) and HeLa cells (right), respectively by flow cytometry after treatment with varying drug concentrations. Optimal HO-1 expression in Vero cells was achieved with 60 μM CoPP at 6 h as previously described, while maximum expression of HO-1 in HeLa cells was achieved with 15 μM CoPP at 14 h after treatment. Data are means ± SEM of three independent experiments. One-way ANOVA, and Tukey's multiple comparison test were used for statistical analyses (^***^*p* < 0.001).

Supplementary Figure 2 HO-1 expression in SH-SY5Y cells treated with CoPP or SnPP and infected with HSV and quantification of virus released into the media. **(A)** Western blot analysis of HO-1 expression in SH-SY5Y cells after treatment with CoPP, SnPP, or vehicle and/or infection with HSV-2 at an MOI 1 for 24 h. **(B)** Flow cytometry analysis of HO-1 expression in SH-SY5Y cells 14 h after treatment with CoPP, SnPP, or vehicle (10 μM) and/or infection with HSV-2 at an MOI 1 for 24 h. C. Quantification of virus plaque forming units (PFU) in the supernatants of SH-SY5Y cells at 6, 12, and 24 h post-infection. PFU determination was done over Vero cells. Data is means ± SEM of three independent experiments. A representative plug is shown for the western blot. One-way ANOVA, and Tukeys's multiple comparison test were used for statistical analyses (^**^*p* < 0.01, ^***^*p* < 0.001).

Supplementary Figure 3 Pharmacological induction of HO-1 activity dampens HSV-encoded gene expression in SH-SY5Y cells. **(A)** Fluorescence microscopy of SH-SY5Y cells treated with HO-1 modulators and infected with a GFP-encoding HSV virus at an MOI 1 (Representatives images are show; 5X magnification). **(B)** Quantification of virus-derived fluorescence in HSV-infected SH-SY5Y cells treated with HO-1 modulating drugs by flow cytometry. **(C)** Viability of SH-SY5Y cells treated with HO-1 modulating drugs before and after infection with HSV at an MOI 1. **(D)** Western blot analyses for HSV proteins gD and VP16 in SH-SY5Y cells at 24 h after infection with HSV-2 at an MOI 1. Representative images are shown for Western blots. One-way ANOVA, and Tukeys's multiple comparison test were used for statistical analyses (^*^*p* < 0.05, ^**^*p* < 0.01).

Supplementary Figure 4 Treatment with a carbon monoxide-releasing molecule reduces HSV-encoded gene expression in SH-SY5Y cells. GFP-derived fluorescence from the HSV-encoded reporter was measured in SH-SY5Y cells treated with CORM-2, 1 h before infection, or inactivated CORM-2 (iCORM-2) at an MOI 1. Data are means ± SEM of three independent experiments. Two-way ANOVA, and Tukey's multiple comparison test were used for statistical analyses (^*^*p* < 0.05, ^***^*p* < 0.001).
