# Supplementary figures and images for: Pharmacological Induction of Heme Oxygenase-1 Impairs Nuclear Accumulation of Herpes Simplex Virus Capsids upon Infection
Source: Front Microbiol. 2017 Oct 31;8:2108. doi: 10.3389/fmicb.2017.02108 (PMC5671570; doi:10.3389/fmicb.2017.02108)

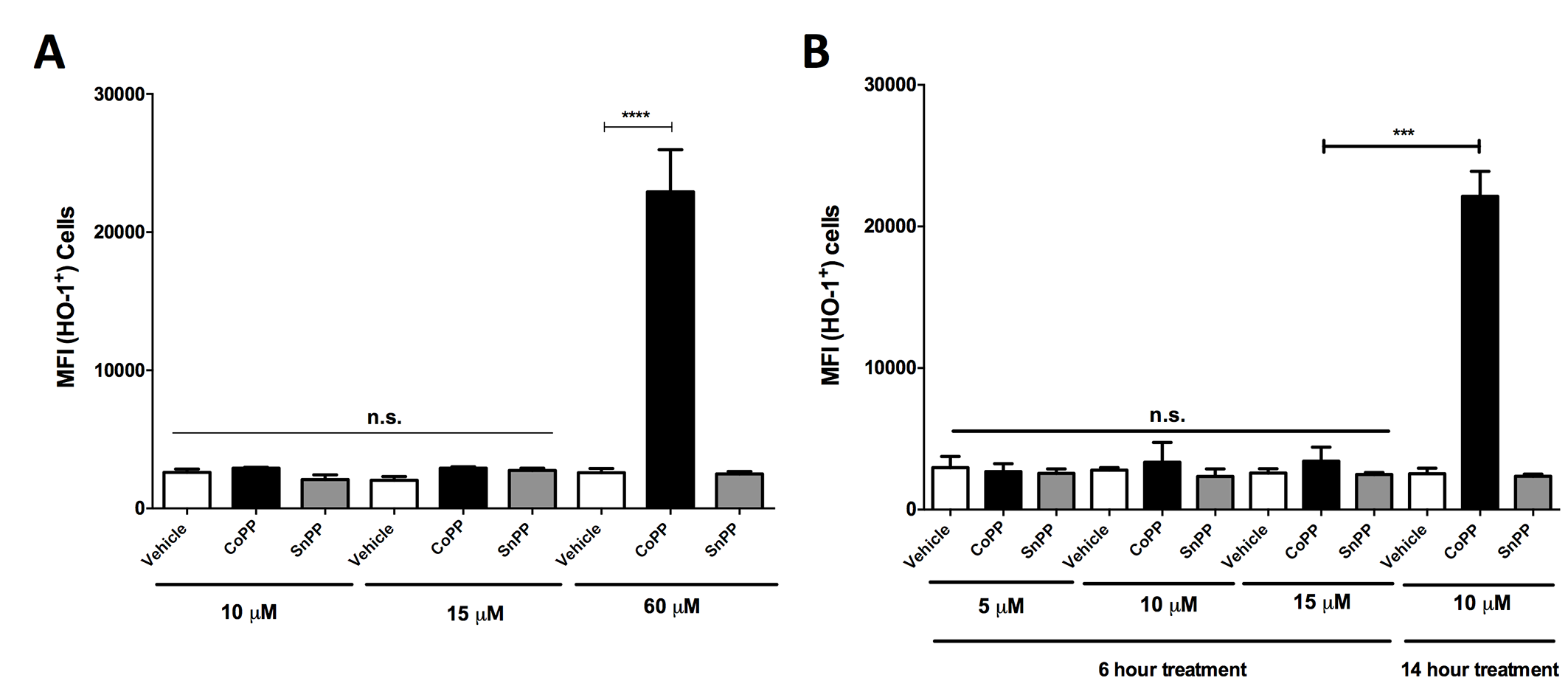

Supplement: Supplementary Figure 1 — HO-1 expression in Vero and HeLa cells treated with low doses of CoPP. (A,B) Quantification of HO-1 expression in Vero (left) and HeLa cells (right), respectively by flow cytometry after treatment with varying drug concentrations. Optimal HO-1 expression in Vero cells was achieved with 60 μM CoPP at 6 h as previously described, while maximum expression of HO-1 in HeLa cells was achieved with 15 μM CoPP at 14 h after treatment. Data are means ± SEM of three independent experiments. One-way ANOVA, and Tukey's multiple comparison test were used for statistical analyses (***p < 0.001). [file Image1.TIFF]

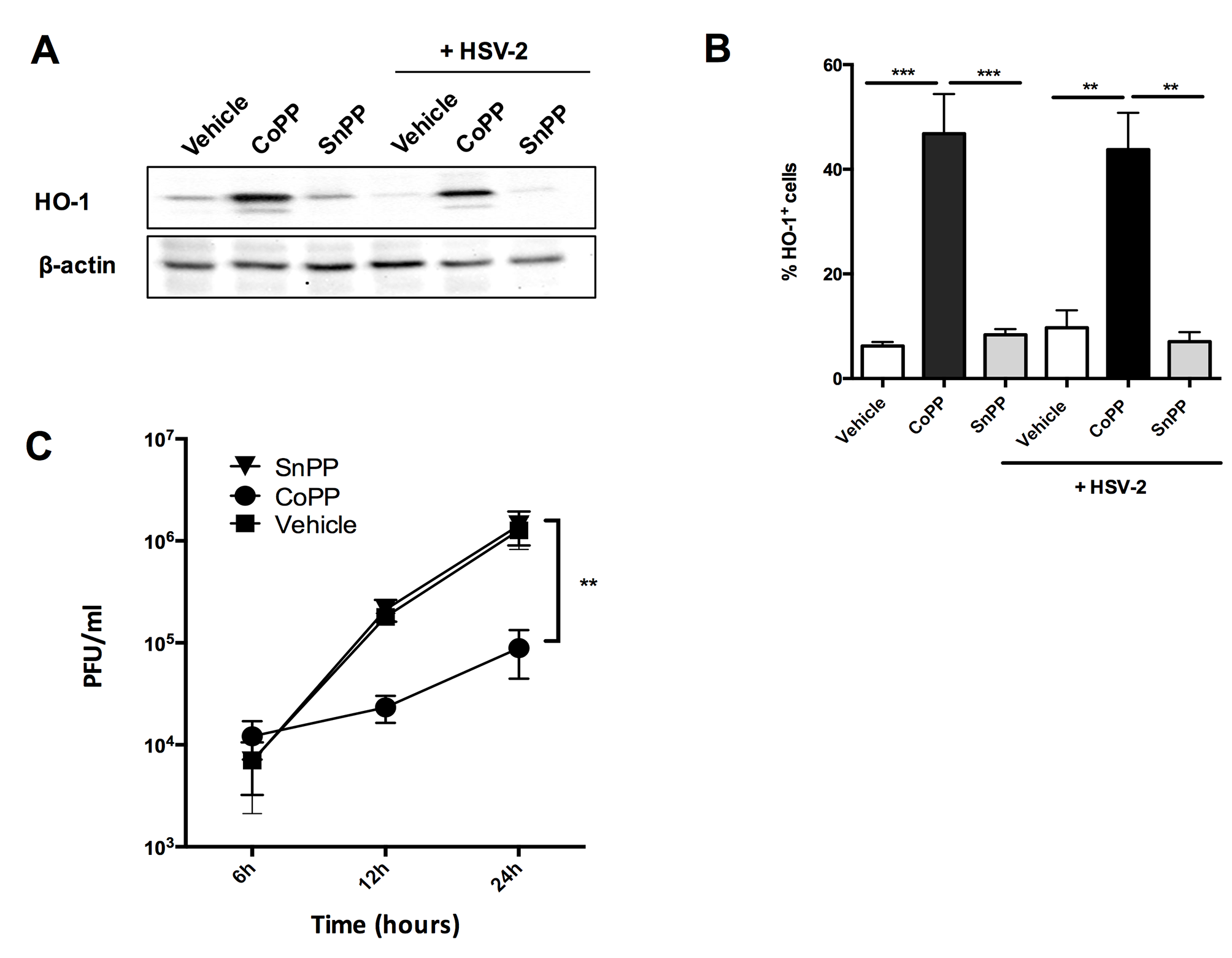

Supplement: Supplementary Figure 2 — HO-1 expression in SH-SY5Y cells treated with CoPP or SnPP and infected with HSV and quantification of virus released into the media. (A) Western blot analysis of HO-1 expression in SH-SY5Y cells after treatment with CoPP, SnPP, or vehicle and/or infection with HSV-2 at an MOI 1 for 24 h. (B) Flow cytometry analysis of HO-1 expression in SH-SY5Y cells 14 h after treatment with CoPP, SnPP, or vehicle (10 μM) and/or infection with HSV-2 at an MOI 1 for 24 h. C. Quantification of virus plaque forming units (PFU) in the supernatants of SH-SY5Y cells at 6, 12, and 24 h post-infection. PFU determination was done over Vero cells. Data is means ± SEM of three independent experiments. A representative plug is shown for the western blot. One-way ANOVA, and Tukeys's multiple comparison test were used for statistical analyses (**p < 0.01, ***p < 0.001). [file Image2.TIFF]

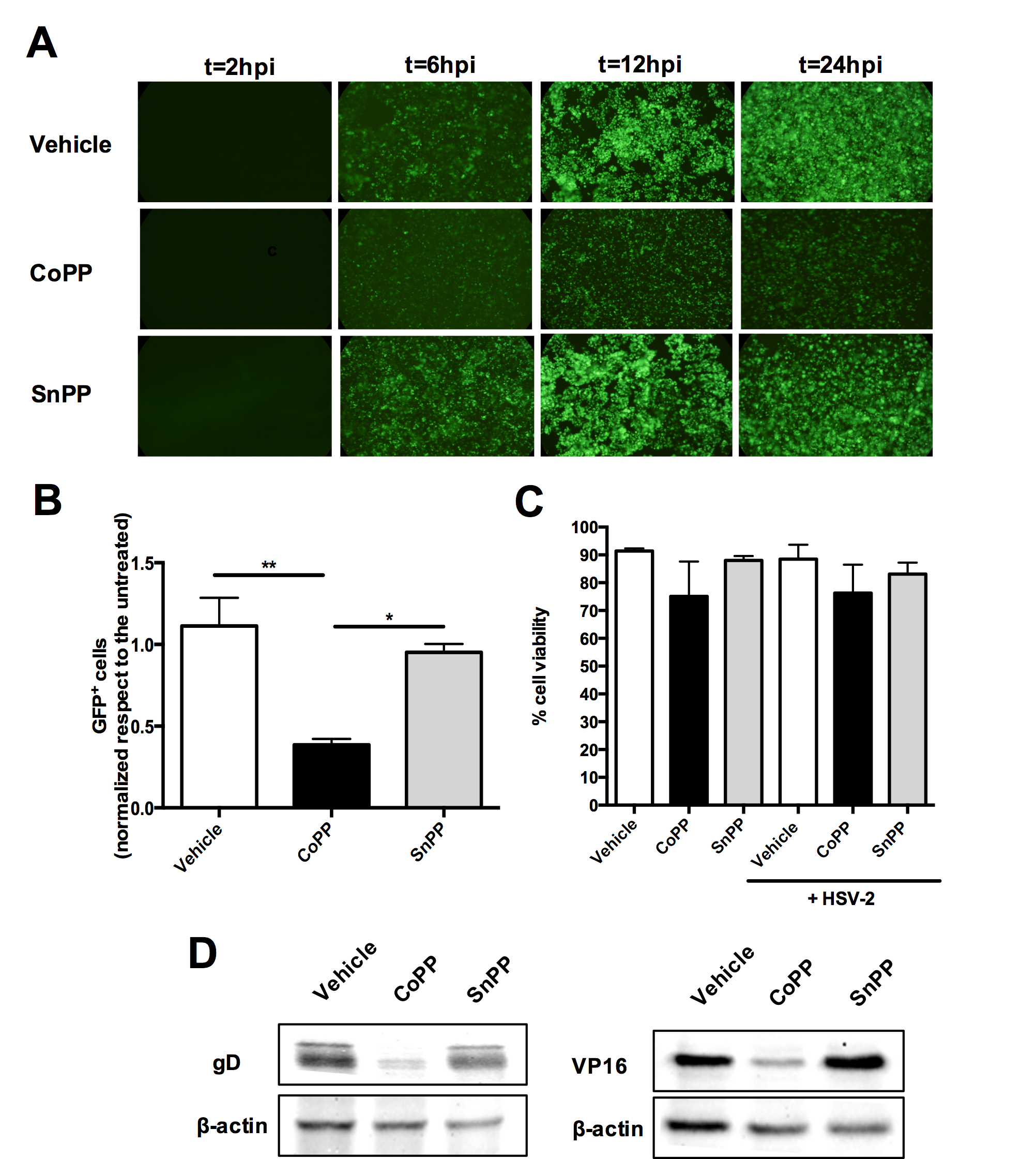

Supplement: Supplementary Figure 3 — Pharmacological induction of HO-1 activity dampens HSV-encoded gene expression in SH-SY5Y cells. (A) Fluorescence microscopy of SH-SY5Y cells treated with HO-1 modulators and infected with a GFP-encoding HSV virus at an MOI 1 (Representatives images are show; 5X magnification). (B) Quantification of virus-derived fluorescence in HSV-infected SH-SY5Y cells treated with HO-1 modulating drugs by flow cytometry. (C) Viability of SH-SY5Y cells treated with HO-1 modulating drugs before and after infection with HSV at an MOI 1. (D) Western blot analyses for HSV proteins gD and VP16 in SH-SY5Y cells at 24 h after infection with HSV-2 at an MOI 1. Representative images are shown for Western blots. One-way ANOVA, and Tukeys's multiple comparison test were used for statistical analyses (*p < 0.05, **p < 0.01). [file Image3.TIFF]

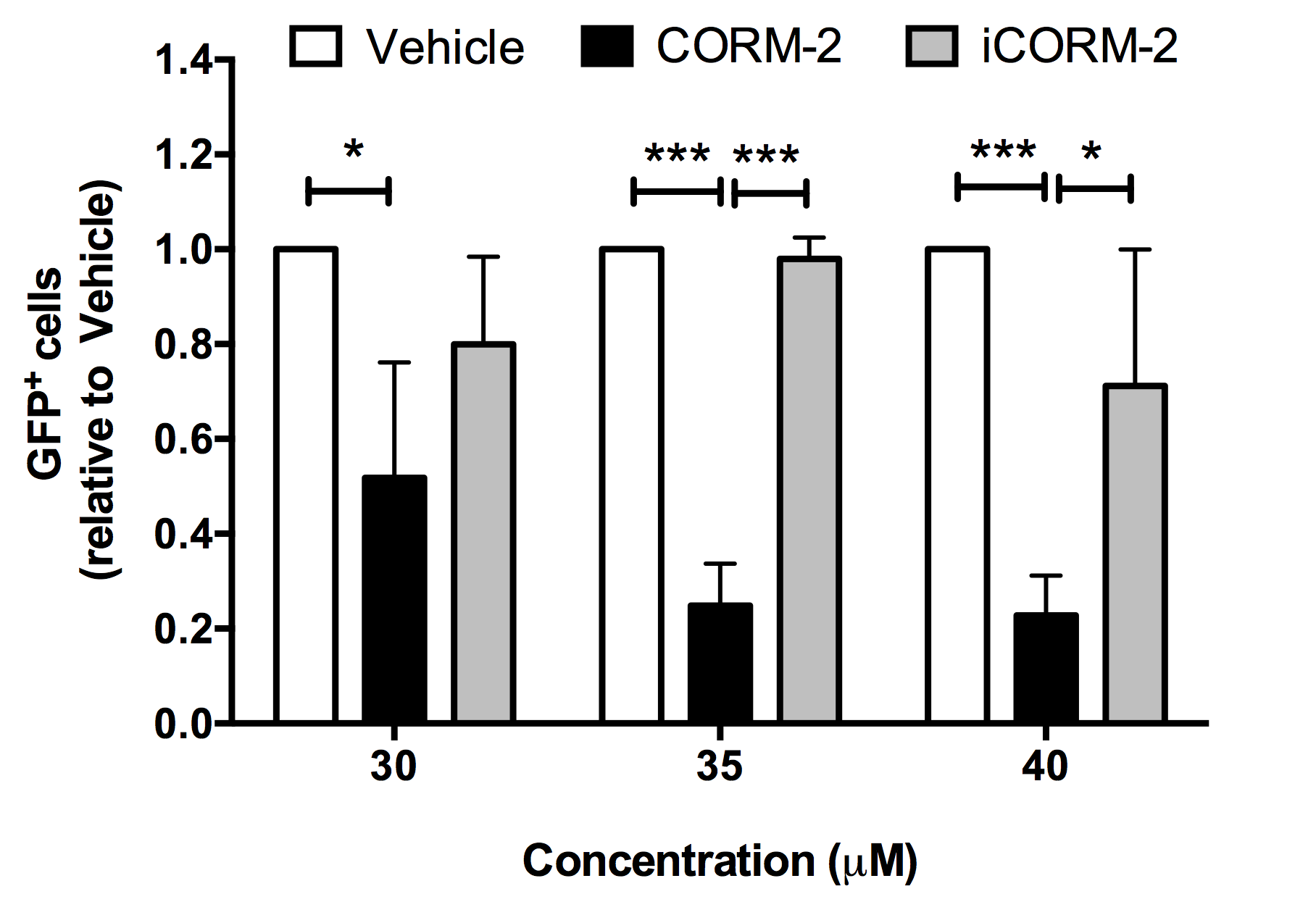

Supplement: Supplementary Figure 4 — Treatment with a carbon monoxide-releasing molecule reduces HSV-encoded gene expression in SH-SY5Y cells. GFP-derived fluorescence from the HSV-encoded reporter was measured in SH-SY5Y cells treated with CORM-2, 1 h before infection, or inactivated CORM-2 (iCORM-2) at an MOI 1. Data are means ± SEM of three independent experiments. Two-way ANOVA, and Tukey's multiple comparison test were used for statistical analyses (*p < 0.05, ***p < 0.001). [file Image4.TIFF]
